# Continuous Cultivation as a Method to Assess the Maximum Specific Growth Rate of Photosynthetic Organisms

**DOI:** 10.3389/fbioe.2019.00274

**Published:** 2019-10-17

**Authors:** Elena Barbera, Alessia Grandi, Lisa Borella, Alberto Bertucco, Eleonora Sforza

**Affiliations:** ^1^Interdepartmental Center Giorgio Levi Cases, University of Padova, Padova, Italy; ^2^Department of Industrial Engineering DII, University of Padova, Padova, Italy

**Keywords:** cyanobacteria, *Anabaena* PCC7122, kinetic model, respirometry, continuous photobioreactors

## Abstract

Modeling the growth of photosynthetic organisms is challenging, due to the complex role of light, which can be limiting because of self-shading, or photoinhibiting in the case of high intensities. A case of particular interest is represented by nitrogen-fixing cyanobacteria, whose growth is controlled not only by the light intensity, but also by the availability of atmospheric nitrogen in the liquid medium. The determination of the maximum specific growth rate is often affected by many variables that, in batch growth systems, may change significantly. On the other hand, in a continuous system, once the steady state is reached the values of all the process variables remain constant, including the biomass concentration and the specific light supply rate. In this work, the diazotrophic cyanobacterium *Anabaena* PCC 7122 was cultivated in continuous photobioreactors, to investigate the role of nitrogen, light and residence time on growth kinetics, and to retrieve the value of the maximum specific growth rate of this organism. In addition, the kinetic parameters for temperature and the half saturation constant for nitrogen (3 mg L^−1^) were measured by respirometric tests. Based on the results of continuous experiments, the specific maintenance rate was found to depend on the light intensity supplied to the reactor, ranging between 0.5 and 0.8 d^−1^. All these parameters were used to develop a kinetic model able to describe the biomass growth in autotrophic conditions. The maximum specific growth rate could hence be determined by applying the kinetic model in the material balances of the continuous photobioreactor, and resulted equal to 8.22 ± 0.69 d^−1^.

## Introduction

Photosynthetic microorganisms, such as microalgae and cyanobacteria, have recently gained researchers' interest due to their great potentialities. For example, they can double their cells even 100 times faster than terrestrial plants (Lam et al., 2012) and require significantly less area to grow with respect to other crops thanks to their high photosynthetic efficiency per unit surface (Darvehei et al., 2018). Moreover, they offer a large number of potential applications: the biomass can be used directly (e.g., in aquaculture), or in environmental applications (e.g., waste water treatment, CO_2_ mitigation and biofuel production), or alternatively high-value compounds can be extracted (e.g., pigments, vitamins) (Fernandes et al., 2015).

Large-scale cultivation of microalgae and cyanobacteria to produce bioproducts and biofuels has greatly increased over the last years (Khan et al., 2018). However, there are still many challenges to face. The most important is represented by the need to increase the process profitability, which is mainly influenced by the biomass productivity. In order to maximize the productivity, the process operating conditions must be optimized and strictly controlled. An important contribution is given by mathematical models that take into account the effect of each process variable (light, temperature, residence time, etc.) for the estimation of key production parameters (biomass growth rate, productivity, etc.) (Darvehei et al., 2018). In addition, they can be used to bridge the existing gap between lab-scale observations, on which prediction of the growth on large scale is based, and the industrial-scale reality (Bernard et al., 2015).

However, modeling the growth of photosynthetic organisms, as well as control and optimization of the process, is challenging, even more than for bacterial or yeast bioprocesses. This is mainly due to higher complexity of their cells and the wide range of mechanisms they use to respond to, or protect themselves from changes in light intensity, temperature and other environmental factors (Bernard et al., 2015).

One of the most important parameters to be determined is the maximum specific growth rate (μ_max_). In general, it is evaluated by cultivating the microorganism in batch systems and subsequently elaborating the experimental data obtained during the exponential phase of growth. However, it is important to notice that the value of μ_max_ determined this way is affected by the experimental conditions, such as light intensity, temperature, pH and medium composition, which are not constant during the growth since biomass, substrates and products concentrations all change exponentially (Stanbury et al., 2016). In particular, the actual light perceived by the cells drops along with the increase of biomass concentration. In this way, the microorganism cannot adapt to the environment in which it is growing, since the adaptation process is not instantaneous (Trilli, 1990). Therefore, it is very difficult to relate “cause and effect” by growing the microorganism in batch systems.

On the other hand, in a continuous system, the growth rate is controlled by the dilution rate (Stanbury et al., 2016) and, once the steady state is reached, the values of all the process variables remain constant, including the biomass and substrate concentrations, and especially the specific light supply rate. Moreover, the adaptation process is no longer a problem because the microorganism is let to adapt to the growing conditions during the transient phase. This allows a quantitative assessment of the effect of the operating variables on the performances of the culture.

In this work, we propose a new approach that combines the use of cultivation in continuous systems and respirometric tests to determine the kinetic parameters of photosynthetic microorganisms' growth (Sforza et al., 2019). In particular, it was applied to study the growth of the nitrogen-fixing cyanobacterium *Anabaena* PCC 7122, which is not only affected by the light intensity, but also by the liquid solubility of atmospheric nitrogen, that controls its availability in the culture medium. The cultivation of diazotrophic cyanobacteria can find promising applications toward the development of a sustainable agriculture (Singh and Datta, 2007), or in the production of valuable pigments, such as phycocyanin (Moreno et al., 1995, 2003), while not requiring nitrogen fertilizers inputs. However, the approach could be applied to other photosynthetic microorganisms as well.

The cyanobacterium was cultivated in continuous photobioreactors at different incident light intensities with and without additional supply of nitrates, in order to investigate the role of nitrogen, together with light and residence time, on growth kinetics. The experimental results obtained were elaborated to retrieve the value of the specific maintenance rate (Gons and Mur, 1980). The kinetic parameters describing the effect of temperature and the half-saturation constant of nitrogen were instead measured by means of respirometric tests. All these parameters were combined in a comprehensive kinetic model able to describe the biomass growth in autotrophic conditions. The maximum specific growth rate μ_max_ could finally be determined by applying the developed kinetic model in the material balances of the continuous reactor, for all the experimental conditions investigated.

## Materials and Methods

### Experimental Strain and Culture Medium

The photosynthetic organism used in this work is *Anabaena* PCC 7122, purchased from the Pasteur Culture Collection of Cyanobacteria, Paris (France). *Anabaena* PCC 7122, also known as *Anabaena cylindrica*, is a diazotrophic filamentous cyanobacterium forming heterocysts (i.e., specific cells where the nitrogen fixation is performed) (Fogg, 1944; Allen and Arnon, 1955), and which does not produce toxins (Phillips and Roberts, 1985; Quiblier et al., 2013). This strain was selected as, in preliminary screening tests, it showed good growth and N-fixation performances compared to other diazotrophic species. The cyanobacterium was maintained in 250 mL Erlenmeyer flasks placed in an orbital shaker, under a continuous light intensity of 75 μmol m^−2^ s^−1^ and ambient temperature. The culture media used in continuous cultivation experiments were modified BG11 and BG11_0_ (i.e., without nitrogen salts), with a final composition as reported in the Supplementary materials (Table S1). In addition, the media were supplied with 2.5 g L^−1^ of NaHCO_3_ to obtain a buffered system with the CO_2_-air gas mixture (section Experimental Setup) and maintain the pH within the optimal interval of 7.5–8.

### Experimental Setup

**Figure 1:**
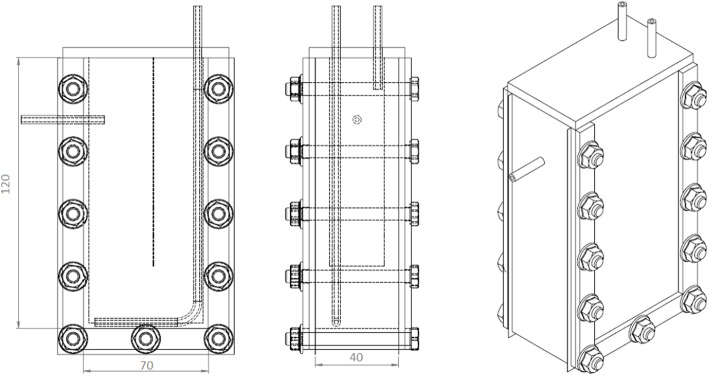
Scheme of the PBR used for the continuous growth experiments. In the first sketch, the dotted vertical line represents the baffle.

All the experiments were carried out in vertical flat-panel polycarbonate photobioreactors having a working volume (*V*_*R*_) of 350 mL and an irradiated surface (*A*_*PBR*_) of 87.5 cm^2^. The photobioreactor (PBR) (Figure 1) was specifically designed for the cultivation of this filamentous cyanobacterium. Since it has a high tendency to aggregate and to form clusters, a thickness of 4 cm was chosen to allow a better stirring of the culture. A thin baffle was inserted to avoid short-circuiting of the inlet medium flow rate, which was fed from the top.

The mixing was ensured by both a stirring magnet placed at the bottom of the reactor and the bubbling of CO_2_-enriched air (5% v/v), sparged from the bottom at a flowrate of 1 L h^−1^. The mixing condition was checked by means of tracer experiments, that allow to consider such a reactor as a completely stirred tank one (CSTR) (Sforza et al., 2014a, 2015). Accordingly, the specific growth rate μ (d^−1^) is equal to the dilution rate *D*, which is the inverse of the residence time τ (d) according to:

(1) $$\begin{array}{l} \mu =D=\frac{1}{\tau } \end{array}$$

Hence, by changing the residence time, different growth rates can be imposed to the culture.

Fresh inlet BG11 or BG11_0_ was continuously fed by means of a tunable peristaltic pump (120S, Watson Marlow, USA), that allows regulating the inlet flow rate Q (mL d^−1^). The reactor volume was kept constant by an overflow tube, from which the biomass was constantly withdrawn. Accordingly, the residence time of the culture inside the PBR was calculated as:

(2) $$\begin{array}{l} \tau =\frac{V_{R}}{Q} \end{array}$$

The temperature was kept constant at T = 24 ± 1°C, in a refrigerated incubator. Continuous artificial white light was provided by a LED lamp (Photon System Instruments, SN-SL 3500-22). The light intensities at the front and back surfaces of the PBR were measured with a photoradiometer (HD 2101.1, Delta Ohm), which quantifies the PAR (Photosynthetically Active Radiation, 400–700 nm).

The reactor was started up in batch operation mode, inoculating the cyanobacterium at initial OD_750_ = 0.3 (corresponding to ~0.1 g L^−1^ dry weight). Once a sufficient biomass concentration was reached (about 1 g L^−1^) the feed peristaltic pump was switched on. When changing any experimental conditions (i.e., residence time or incident light intensity), a transient period was observed (7–10 d), after which a stable steady-state was reached. For each condition, steady state was maintained for at least 5 days, during which biomass samples were taken twice a day, and the corresponding experimental values were averaged accordingly.

### Analytical Procedures and Calculations

The biomass concentration in the PBR was monitored daily by both optical density at 750 nm (OD_750_) and dry weight (*c*_*x*_, g L^−1^) measurements. OD_750_ was measured with a UV-Visible double beam spectrophotometer (UV1900, by Shimadzu, Japan). The dry weight was determined by filtering 10 mL of culture sample on previously dried 0.45 μm nitrocellulose filters. The filters were then dried at 105°C in a laboratory oven for at least 2 h. The biomass volumetric and areal productivities were then calculated according to:

(3) $$\begin{array}{l} P_{x,V}\left(\frac{g}{L\cdot d}\right)=\frac{c_{x}}{\tau } \end{array}$$

Once the steady-state was reached, the number of heterocysts was experimentally counted with a Bürker chamber, in order to compare their concentration when the cyanobacterium was grown with or without nitrates feed. Moreover, at steady-state, the experimental values of the specific light supply rate *I*_*sp*_ (i.e., the amount of light supplied per unit mass of biomass and unit time) were determined according to:

(4) $$\begin{array}{l} I_{sp}\left(\frac{mmol}{g\cdot d}\right)=\frac{I_{0}}{C_{x}\;W} \end{array}$$

where *I*_0_ is the incident light intensity at the front of the PBR and *W* is the PBR width (0.04 m).

### Kinetic Model Description

According to the result of elemental analysis performed on dried biomass of *Anabaena* PCC 7122, its chemical formula was determined and, thus, the corresponding autotrophic growth stoichiometry could be written as follows:

$$\begin{array}{l} 0.2518{\;H}_{2}O+0.0202{\;N}_{2}+0.2314{\;CO}_{2}\\ \;+0.0018\;H_{2}PO_{4}^{-}+0.0018\;HPO_{4}^{2-}\\ \;\to \;C_{0.2314}H_{0.5036}O_{0.2210}N_{0.0404}P_{0.0036}\\ \;+0.2513{\;O}_{2}+0.0054\;OH^{-} \end{array}$$

According to photosynthesis and nitrogen-fixation processes, water, atmospheric nitrogen, carbon dioxide and phosphates are consumed to produce new biomass and release oxygen.

The equation describing the autotrophic microalgal growth rate (*r*_*x*_, g L^−1^ d^−1^) can be written as a function of biomass concentration (*c*_*x*_, g L^−1^), the maximum specific growth rate (μ_max_, d^−1^), temperature (Φ(*T*)), specific light (*f*(*I*_*sp*_)) and the most limiting nutrient that, in this case, is nitrogen (*f*(*N*)). Moreover, the kinetic model takes into account the specific maintenance rate (μ_*e*_), i.e., the loss of biomass due to turnover of cellular components and cell repair, which represents a negative term, thus reducing the net biomass growth rate, according to Equation (5):

(5) $$\begin{array}{l} r_{x}=c_{x}\cdot \mu_{\max}\cdot \Phi \left(T\right)\;\cdot \;f\left(N\right)\;\cdot \;f\left(I_{sp}\right)-\mu_{e}\;\cdot \;c_{x} \end{array}$$

The term accounting for the effect of temperature was calculated according to the model proposed by Bernard et al. (2015) based on the so-called cardinal temperature model with inflection (CTMI) (Rosso et al., 1993):

(6) $$\begin{array}{l} \mu \left(T\right)=\{\begin{array}{ll} 0 & \text{for }T<T_{\min}\\ \mu_{opt}\cdot \Phi (T) & \text{for }T_{\min}<T<T_{\max}\\ 0 & \text{for }T>T_{\max} \end{array} \end{array}$$

where

(7) $$\begin{array}{l} \Phi \left(T\right)=\frac{\left(T-T_{\max}\right){\left(T-T_{\min}\right)}^{2}}{\begin{array}{c} \left(T_{opt}-T_{\min}\right)[\left(T_{opt}-T_{\min}\right)\left(T-T_{opt}\right)\\ -\left(T_{opt}-T_{\max}\right)\left(T_{opt}+T_{\min}-2T\right)] \end{array}} \end{array}$$

The function Φ(*T*) includes three parameters with a physical meaning: *T*_min_, *T*_max_ and *T*_*opt*_ (°C). *T*_min_ and *T*_max_ are the temperatures below and above which there is no growth nor respiration, while *T*_*opt*_ is the temperature at which the growth rate is maximum. In this way, μ(*T*) assumes a typical bell-shaped profile.

The term accounting for the limitation due to the nitrogen concentration (*c*_*N*_) was represented as a Monod function (Monod, 1949):

(8) $$\begin{array}{l} f\left(N\right)=\frac{c_{N}}{K_{N}+c_{N}} \end{array}$$

where *K*_*N*_ is the half-saturation constant (mg_*N*_ L^−1^).

The term accounting for the effect of light was modeled according to Haldane (1930), in order to consider both the saturation and photoinhibition effects:

(9) $$\begin{array}{l} f\left(I_{sp}\right)=\frac{I_{sp}}{K_{L}+I_{sp}+\frac{{I_{sp}}^{2}}{K_{I}}} \end{array}$$

*K*_*L*_ and *K*_*I*_ are the half-saturation and the inhibition constants (mmol photons g^−1^ d^−1^) of light, respectively, expressed in terms of specific light supply rate (*I*_*sp*_, mmol photons g^−1^ d^−1^) in order to account for self-shading effects due to increasing biomass concentration, according to Equation (4).

The specific maintenance rate (μ_*e*_), which was first described for heterotrophic microorganisms by Pirt (1965) as “the energy consumed for functions other than productions of new cell material,” was calculated according to the model proposed by Gons and Mur (1980). These authors observed that growth yields decrease at low growth rates as a consequence of a requirement of energy for maintenance of existing cells. Therefore, the growth rate is proportional to the energy absorbed, with the exception of the energy required for the cell maintenance. According to these considerations and to the energy balance, they derived the following equation:

(10) $$\begin{array}{l} \mu =\left(\frac{dE}{dt}\;\cdot \;\frac{1}{X}\right)\;\cdot \;c-\mu_{e} \end{array}$$

where $\frac{dE}{dt}$ is the light energy uptake rate (J d^−1^), *X* is the energy stored in the culture biomass (J), and *c* is the “true” efficiency of light conversion into the chemical energy that is stored in biomass (dimensionless). Gons and Mur (1980) applied the model to the continuous light-limited growth of *Scenedesmus protuberans* and found a linear dependence of the growth rate on the specific light uptake rate $\left(\frac{dE}{dt}\cdot \frac{1}{X}\right)$. Hence, by correlating their data according to Equation (10), they derived *c* and μ_*e*_values.

In our case, the specific light uptake rate $\frac{dE}{dt}\cdot \frac{1}{X}$ (d^−1^), which represents the amount of light energy utilized per unit of biomass over time, was hence calculated from the experiments according to:

(11) $$\begin{array}{l} \frac{dE}{dt}\cdot \frac{1}{X}=\frac{I_{abs}\cdot E_{p}\cdot A_{PBR}}{c_{x}\cdot LHV\cdot V_{R}} \end{array}$$

where *I*_*abs*_ is the absorbed photon flux density, calculated by subtracting the irradiance at the back of the PBR from the incident light intensity (μmol photons m^−2^ s^−1^), *E*_*p*_ is the average energy of a photon (assumed to be 0.223 kJ/mmol), *A*_*PBR*_ is the illuminated surface of the PBR (m^2^), *LHV* is the lower heating value of the biomass (assumed to be equal to 18.66 kJ/g Zaimes and Khanna, 2013) and *V*_*R*_ is the PBR volume (m^3^).

### Respirometric Tests

In order to retrieve the values of the parameters contained in Equations (7–9), respirometric tests were carried out. The experimental apparatus and protocol used were the same described by Sforza et al. (2019).

This technique has already been used to determine kinetic parameters of microalgae growth by Decostere et al. (2013) and to evaluate microalgal performances in wastewater by Rossi et al. (2018). The protocol is based on the measurement of oxygen production or consumption due to the growth and respiration of microalgal biomass, to which it is correlated trough yields factors:

(12) $$\begin{array}{l} Y_{O_{2}/x}=\frac{\frac{dc_{O_{2}}}{dt}}{\frac{dc_{x}}{dt}}=\frac{OPR}{r_{x}} \end{array}$$

where *Y*_*O*_2_/*x*_ is the oxygen/biomass yield and *OPR* is the oxygen production rate (mg_O2_ L^−1^ d^−1^). It follows that the normalized oxygen production rate (*OPR*_*sp*_ = *OPR*/*c*_*x*_) is:

(13) $$\begin{array}{l} \frac{OPR}{c_{x}\;}=\mu_{\max}\;\cdot \Phi \left(T\right)\cdot \;f\left(N\right)\cdot f\left(I_{sp}\right)\cdot Y_{O_{2}/x}-\mu_{e}\cdot Y_{O_{2}/x} \end{array}$$

Each respirometric test started by preparing a cyanobacterium inoculum of about 0.2 g L^−1^ of DW. After having filled a flask with it, the inoculum was exposed to light-dark cycles of 5:5 min each, obtained by means of a digital controller connected to a LED lamp. The concentration of nitrogen (supplied as sodium nitrate) or the experimental conditions (light intensity and temperature) were varied independently to study the effect of these variables on the growth. Each test lasted about 3 h and resulted in dissolved oxygen (DO) concentration profiles along with time. During light phases, a positive increase of DO is measured (i.e. OPR), while a negative one is observed during dark phases (i.e., OCR, oxygen consumption rate).

Experimental DO data, obtained from the oximeter (HD2109.1 DELTA OHM), were corrected by taking into account the occurrence of oxygen mass transfer with the atmosphere and, then, fitted by a straight line.

(14) $$\begin{array}{l} \frac{dC_{O_{2}}}{dt}=k_{L}a\left(C_{O_{2}}^{*}-C_{O_{2}}\right)+OPR \end{array}$$

Where $\frac{dC_{O_{2}}}{dt}$ is the time derivative of DO concentration (mg_O2_ L^−1^ d^−1^) that corresponds to the slope of the straight line, *k*_*L*_*a* is the global oxygen mass transfer coefficient that was determined experimentally by Sforza et al. (2019) and is equal to 0.0033 min^−1^, $C_{O_{2}}^{*}$ is the oxygen saturation concentration in the liquid (mg_O2_ L^−1^) and *C*_*O*_2__is the DO concentration (mg_O2_ L^−1^).

At least four measurements were performed and averaged to obtain the OPR value of each condition (i.e., four dark-light cycles of 5:5 min each). The specific OPR_sp_ and OCR_sp_ (mg_O2_ ${\text{mg}}_{\text{X}}^{-1}$ d^−1^) were then obtained by normalization of OPR and OCR (mg_O2_ L^−1^ d^−1^) with respect to the initial biomass concentration measured as dry weight (mg_X_ L^−1^).

The value of OPR_sp_ obtained during the light phase represents a net oxygen production rate, because it includes the oxygen consumption, which still occurs even under light exposure. This consumption represents energy loss that is used for maintenance of biological functions, which is mathematically described by the specific rate of maintenance energy μ_*e*_ (Equation 5). Accordingly, by summing the absolute values of OCR_sp_ to OPR_sp_, the actual photosynthetic growth is obtained. Mathematically, this translates to:

(15) $$\begin{array}{l} \frac{OPR}{c_{x}\;}+\left|\frac{OCR}{c_{x}\;}\right|=\mu_{\max}\;\cdot \Phi \left(T\right)\cdot \;f\left(N\right)\cdot f\left(I_{sp}\right)\cdot Y_{O_{2}/x} \end{array}$$

The experiments were performed so that all the operating conditions, except one, were set to their optimal values. In this way, it is possible to study the effect of a single variable. For example, to study the effect of the nitrogen concentration, temperature and light intensities were set to their optimal values and Equation (15) became:

(16) $$\begin{array}{l} \frac{OPR}{c_{x}\;}+\left|\frac{OCR}{c_{x}\;}\right|=\left(\mu_{\max}\cdot Y_{O_{2}/x}\right)\cdot \frac{c_{N}}{K_{N}+c_{N}} \end{array}$$

The same is valid when studying the effects of temperature and light. In these cases, nitrogen was supplied in a non-limiting concentration.

### μ_max_ Estimation

The respirometric tests allowed to evaluate all the parameters included in Equations (7–9) (*K*_*N*_, *K*_*L*_, *K*_*I*_, *T*_min_, *T*_*opt*_, *T*_max_), whereas elaborating the experimental data obtained from continuous cultivation with the model of Gons and Mur (1980) allowed to calculate the value of μ_*e*_. Therefore, μ_max_ is the only unknown kinetic parameter in Equation (6) that needs to be estimated.

From the material balance of a CSTR:

(17) $$\begin{array}{l} \frac{dc_{x}}{dt}=\frac{c_{x,in}}{\tau }-\frac{c_{x}}{\tau }+r_{x} \end{array}$$

at steady state ($\frac{dc_{x}}{dt}=0$) and with null inlet biomass concentration, *r*_*x*_ can be calculated by simply dividing the steady-state biomass concentration measured experimentally by the residence time (τ):

(18) $$\begin{array}{l} r_{x}=\frac{c_{x}}{\tau } \end{array}$$

By equaling Equation (18) with Equation (5), i.e., comparing the values of *r*_*x*_ obtained at the different experimental conditions with the kinetic model, the only unknown variable is μ_max_, which can hence be calculated as:

(19) $$\begin{array}{l} \mu_{\max\;}=\frac{1+\mu_{e}\cdot \tau }{\;\tau \cdot \phi \left(T\right)\cdot \frac{c_{N}}{K_{N}+c_{N}}\cdot \frac{I_{sp}^{\exp}}{I_{sp}^{\exp}+K_{L}+\frac{{\left(I_{sp}^{\exp}\right)}^{2}}{K_{I}}}\;} \end{array}$$

where *c*_*N*_ is the experimental concentration of nitrogen (mg_N_ L^−1^).

### Statistical Significance

*T*-student tests were applied to ascertain statistical differences in biomass concentration and productivity obtained under cultivation in BG11 and BG11_0_. The level of statistical significance was assumed for *p-value* < 0.05.

## Results and Discussion

### Continuous Cultivation

Continuous experiments were carried out to assess the effect of operating variables on biomass productivity. The effect of residence time, which is the main operating variable in continuous photobioreactors, was investigated (τ = 1–4.6 d), under constant light intensities of 190 μmol m^−2^ s^−1^ and of 650 μmol m^−2^ s^−1^, by feeding BG11_0_ medium, i.e., with no nitrogen salts. In addition, *Anabaena* was also cultivated under a constant light intensity of 650 μmol m^−2^ s^−1^ by feeding BG11 medium with 3 g L^−1^ of sodium nitrate, in order to verify if the concentration of atmospheric nitrogen dissolved in the liquid, determined by its solubility, was limiting the growth of the cyanobacterium.

**Figure 2:**
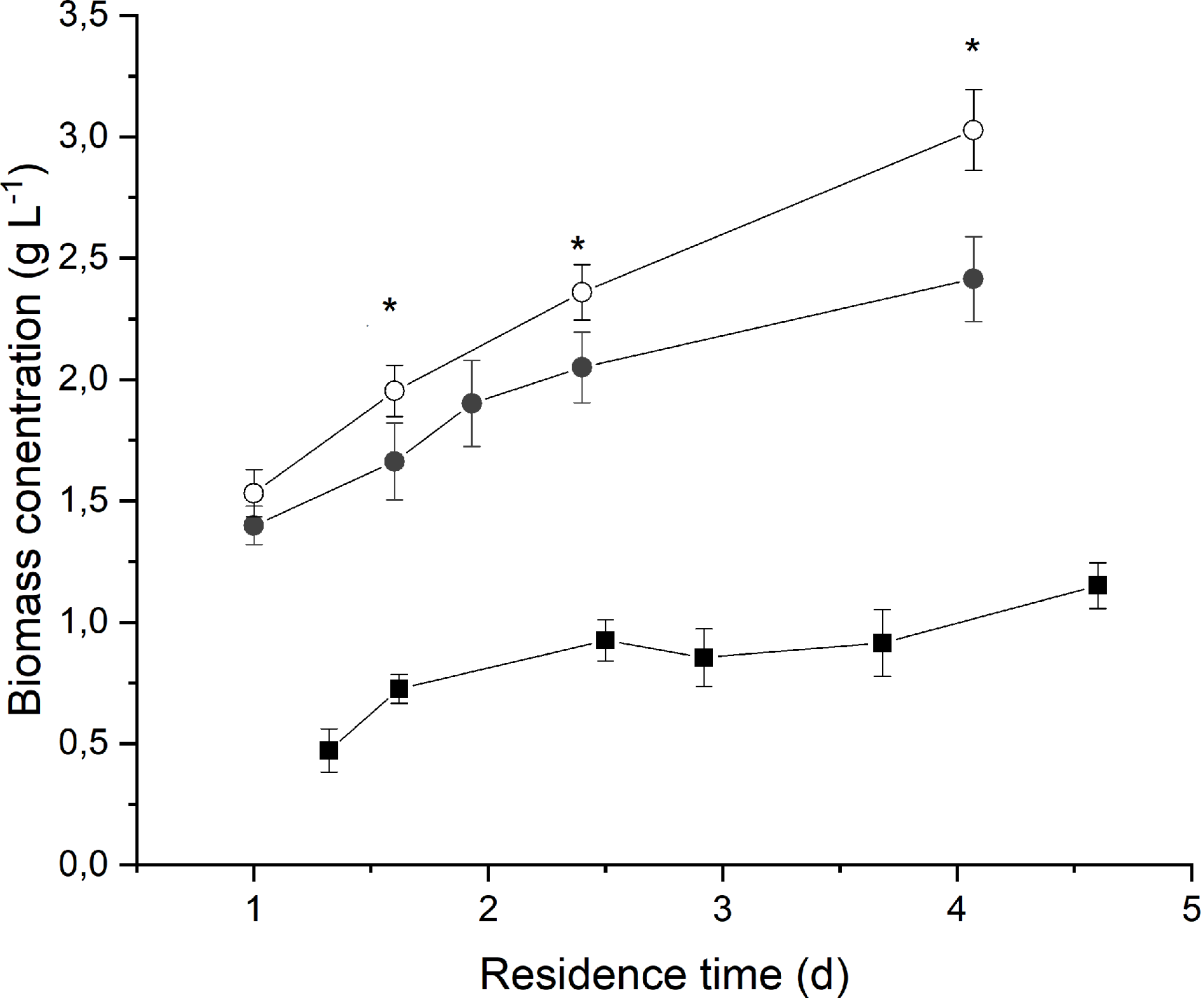
Biomass concentration as a function of residence time at 190 μmol m^−2^ s^−1^ and BG11_0_ medium (full squares) 650 μmol m^−2^ s^−1^ and BG11_0_ medium (full circles), and 650 μmol m^−2^ s^−1^ and BG11 medium (open circles). Asterisks mark statistically different results between cultivation in BG11 and BG11_0_.

Figure 2 reports the average biomass concentration (g L^−1^) obtained at steady-state for each value of residence time at different irradiances and medium fed. It can be seen that it increased along with the residence time, as expected. However, the observed trend is not linear, and it tends to flatten at high residence times as a consequence of self-shading effects between cells (Janssen et al., 2003; Sforza et al., 2014b). Moreover, a higher incident light intensity resulted in an increased biomass concentration, suggesting that, at 190 μmol m^−2^ s^−1^, the growth of *Anabaena* was limited by light.

The comparison between biomass concentrations obtained when supplying nitrates with respect to the ones obtained under strictly nitrogen-fixing conditions highlights that both optical density and dry weight are slightly higher in the case of biomass cultivated in presence of 3 g L^−1^ of sodium nitrate. However, the difference is not considerable, except for the higher residence time. This suggests that *Anabaena*'s growth is not significantly limited by nitrogen concentration or uptake even under atmospheric nitrogen-fixing conditions.

By comparing the heterocysts concentration (number of cells per μg of dry weight) in the reactors irradiated at 650 μmol m^−2^ s^−1^ as expected, the value resulted higher in the case of biomass cultivated in nitrogen-fixation conditions, for all the residence times studied (Figure S1). However, the presence of heterocysts was noticed also in the case of biomass cultivated with a directly available nitrogen source (NaNO_3_) in the culture medium.

**Figure 3:**
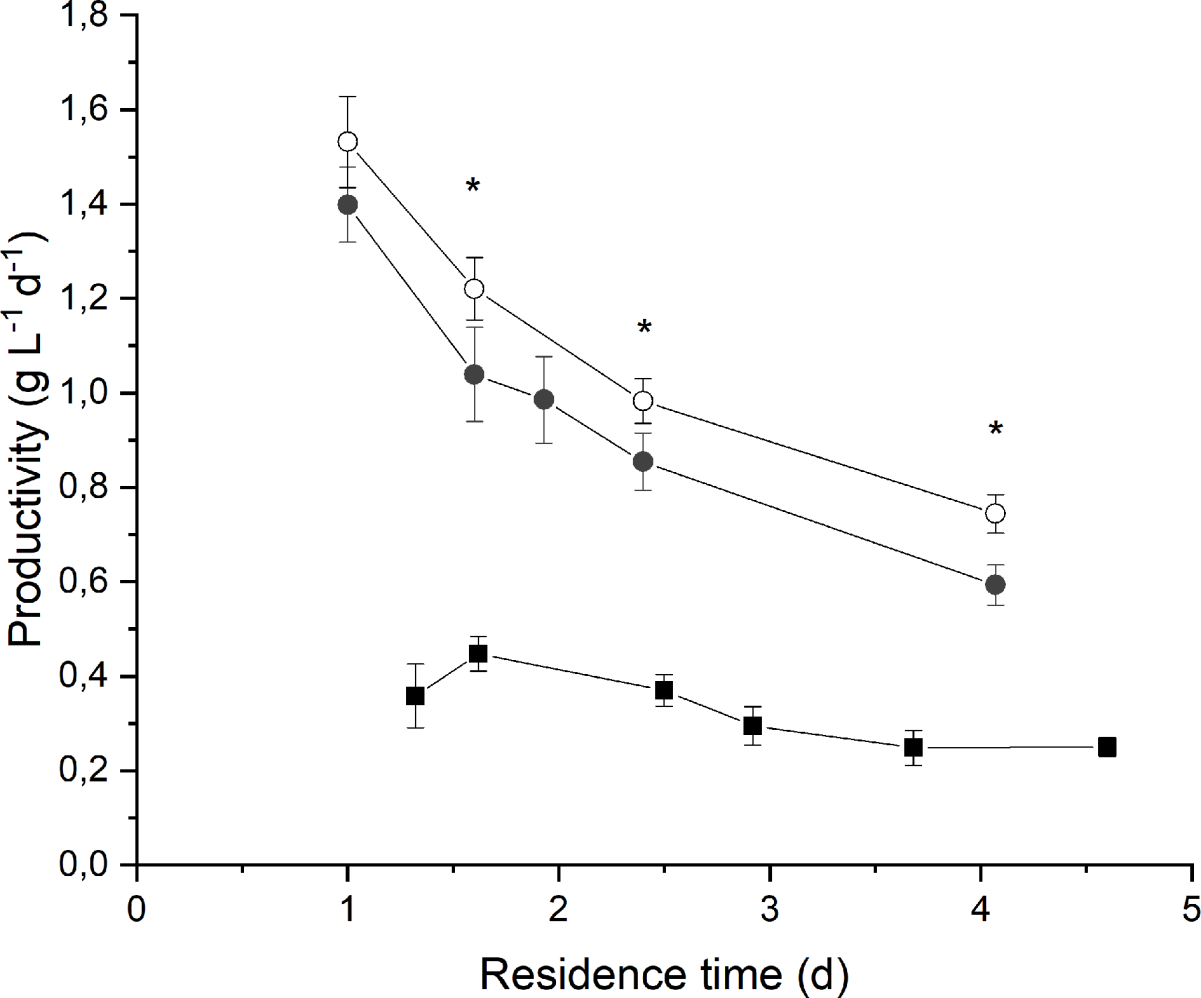
Biomass volumetric productivity as a function of residence time at 190 μmol m^−2^ s^−1^ and BG11_0_ medium (full squares) 650 μmol m^−2^ s^−1^ and BG11_0_ medium (full circles), and 650 μmol m^−2^ s^−1^ and BG11 medium (open circles). Asterisks mark statistically different results between cultivation in BG11 and BG11_0_.

By observing Figure 3 it is evident that biomass volumetric productivity increased if the residence time decreased, so that simultaneously the biomass growth rate increased. It is widely acknowledged that when considering continuous autotrophic cultivation of photosynthetic microorganisms in a chemostat reactor, the biomass productivity profile shows a maximum in correspondence of the optimal value of residence time. On the right-hand side, the biomass growth is limited by self-shading effect along the reactor depth; on the left-hand side, when increasing the volumetric flow rate, the biomass removal from the reactor overcomes the biomass growth until the wash-out residence time is reached (Moreno et al., 2003; Takache et al., 2010; Barbera et al., 2015). Clearly, the optimum operating residence time depends on the incident irradiance, and should be assessed accordingly. In fact, in the case of an incident light intensity equal to 190 μmol m^−2^ s^−1^, the maximum of productivity (0.45 ± 0.04 g L^−1^ d^−1^) was reached for a residence time of about 1.6 d. Instead, at 650 μmol m^−2^ s^−1^ the maximum was not reached even if the residence time was decreased down to 1 d, suggesting that an even higher biomass growth rate could have been applied. However, a remarkable value of volumetric biomass productivity (1.4 ± 0.1 g L^−1^ d^−1^) was found at 1 d of residence time when irradiating the photobioreactor with 650 μmol m^−2^ s^−1^. Reported productivity values of *Anabaena* ATCC 33047 obtained under outdoor cultivation conditions range from 0.06 g L^−1^ d^−1^ in the winter to 0.13 g L^−1^ d^−1^ in the summer season (Moreno et al., 2003).

### Maintenance Energy

**Figure 4:**
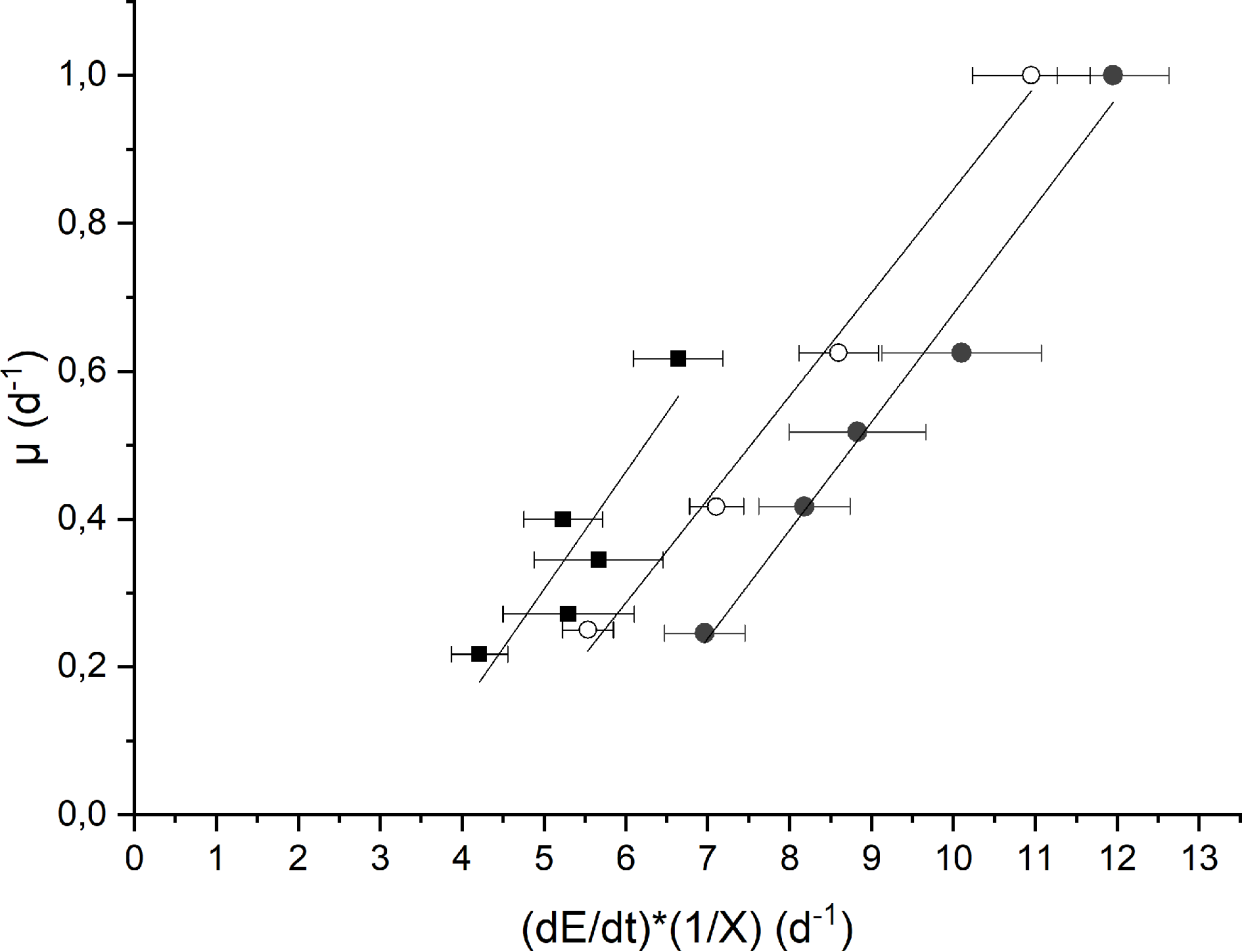
Plot of the growth rate as a function of the specific light supply at 190 μmol m^−2^ s^−1^ and BG11_0_ medium (full squares) 650 μmol m^−2^ s^−1^ and BG11_0_ medium (full circles), and 650 μmol m^−2^ s^−1^ and BG11 medium (open circles).

The evaluation of the maintenance energy requirement in *Anabaena* PCC 7122 was obtained based on the elaboration of experimental data of continuous cultivation at low incident light (*I*_*in*_ = 190 μmol m^−2^ s^−1^) as well as at high light (*I*_*in*_ = 650 μmol m^−2^ s^−1^), both for the growth in presence of nitrates and not, according to the model described in section Kinetic Model Description. The results are reported in Figure 4.

Each data set was fitted according to Equation (10) to obtain the values of the “true” efficiency of light conversion into the chemical energy stored in biomass (*c*) (i.e., the slope of the straight line) and the specific maintenance energy (i.e., the intercept). The values of the regressed parameters and the quality of the fitting are summarized in Table 1.

**Table 1:** Parameters of linearization of data reported in Figure 4, according to Equation (10).

	***I_***o***_*(μmol m^**−2**^ s^**−1**^)**	***c* (-)**	***μ_*E*_*(d^−1^)**	***R*^***2***^**
BG11_0_	190	0.16 ± 0.04	0.49 ± 0.24	0.81
	650	0.15 ± 0.01	0.79 ± 0.11	0.98
BG11	650	0.14 ± 0.01	0.55 ± 0.07	0.99

The results obtained are comparable to the values reported by Gons and Mur (1980). In fact, they obtained a value of *c* that varies between 0.11 and 0.13, which is very similar to our values (0.14–0.16).

On the other hand, by observing the values of μ_*E*_, it can be seen that a higher incident light intensity corresponded to a higher value of specific maintenance rate. For the biomass cultivated in BG11_0_, a value of 0.49 d^−1^ corresponded to the low incident light of 190 μmol m^−2^ s^−1^, whereas 0.79 d^−1^ to the higher one (650 μmol m^−2^ s^−1^). This suggests that, at 650 μmol m^−2^ s^−1^, a large part of energy was diverted from the biomass growth to cell repair, possibly due to photoinhibition phenomena (Kliphuis et al., 2012).

In addition, it appears that at high light intensity (650 μmol m^−2^ s^−1^), the specific maintenance rate was slightly lower for the biomass cultivated in presence of sodium nitrate. This is reasonable because the process of atmospheric nitrogen fixation is undoubtedly energy demanding.

Summarizing, our results suggest that irradiation intensity affects the value of the maintenance rate μ_*E*_, according to what already observed also for the microalgae *Scenedesmus obliquus* by Sforza et al. (2015). In particular, μ_*E*_ was observed to be strongly increased under inhibiting irradiation. On the contrary, Gons and Mur (1980) found a μ_*E*_ independent from the incident light intensity used. However, this can be explained by considering that these authors performed all the experiments at irradiances below the inhibiting value. In fact, other authors (Kliphuis et al., 2012) found that higher light intensities resulted in lower biomass/light yields, suggesting that indeed a larger portion of the incident light was “wasted.”

### Results of Respirometric Tests

A series of respirometric tests were performed with the aim of retrieving the kinetic parameters to describe the growth of *Anabaena* PCC 7122 in autotrophic conditions. In the following sections, the results obtained for the light, temperature and nitrogen effects are reported and discussed.

#### Light Effect

The effect of light on growth kinetics was investigated, since it is the main source of energy, essential to support metabolism in autotrophic conditions (Yun and Park, 2003).

The incident light intensity was changed every 4 light-dark cycles and progressively set to values of 50, 75, 100, 150, 450, and 730 μmol m^−2^ s^−1^.

**Figure 5:**
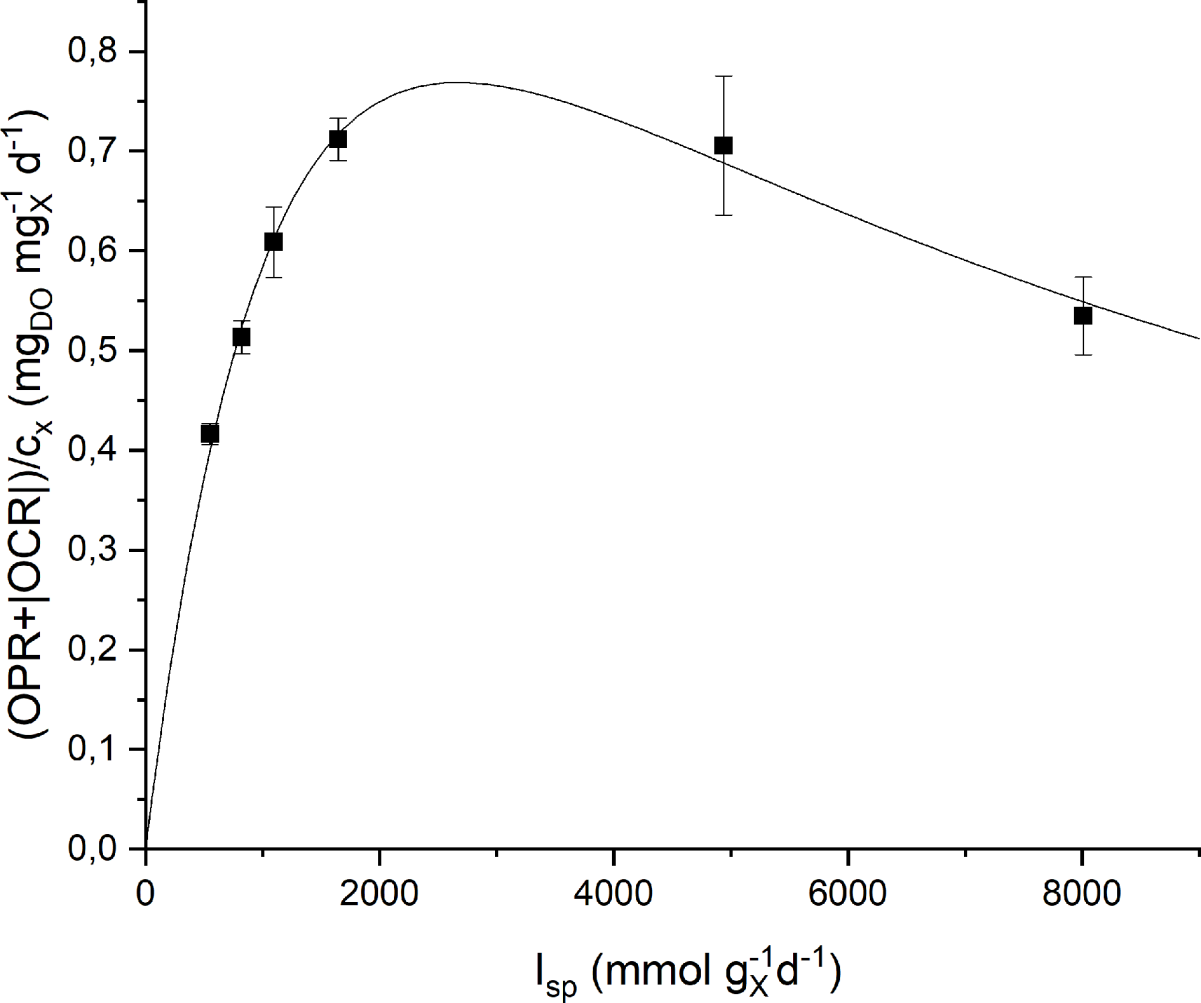
Specific oxygen production rate as a function of specific light supply rate: experimental data (dots) and fitted Haldane model (continuous line).

Respirometric tests were carried out with two different preinocula: one adapted to a low incident light of 150 μmol m^−2^ s^−1^, the other adapted to a high incident light of 650 μmol m^−2^ s^−1^. The results obtained experimentally were plotted as a function of the specific light supply rate (*I*_*sp*_) and then fitted with the Haldane model (Equation 9). Figure 5 shows the experimental data together with the fitting curves for the low light-adapted preincoulum (the high light-adapted one showed a similar trend). As it can be observed, the biomass growth rate, which is correlated to the sum of oxygen production and consumption rates (Equation 15), increased with increasing values of *I*_*sp*_ up to a maximum value, and then decreased due to inhibition effects.

The quality of fitting is good and the obtained model parameters together with their standard deviations are summarized in Table 2. It can be observed that the values of the half-saturation and inhibition constants are very similar in the two cases, suggesting the reliability of the proposed protocol to assess the effect of light intensity on the growth kinetics, regardless the previous inoculum acclimation.

**Table 2:** Summary of the fitted parameters of the Haldane model.

**Adaptation**	***K*_**L**_ (mmol photons ${\text{g}}_{\text{x}}^{-1}$ d^**−1**^)**	***K*_***L***_ (mmol photons ${\text{g}}_{\text{x}}^{-1}$ d^**−1**^)**
Low light	2,023 ± 515	3,531 ± 958
High light	2,192 ± 575	3,730 ± 1,295

#### Temperature Effect

Besides light intensity, temperature is the most important factor influencing the microorganism growth in autotrophic and nutrient unlimited conditions (Bernard and Rémond, 2012).

**Figure 6:**
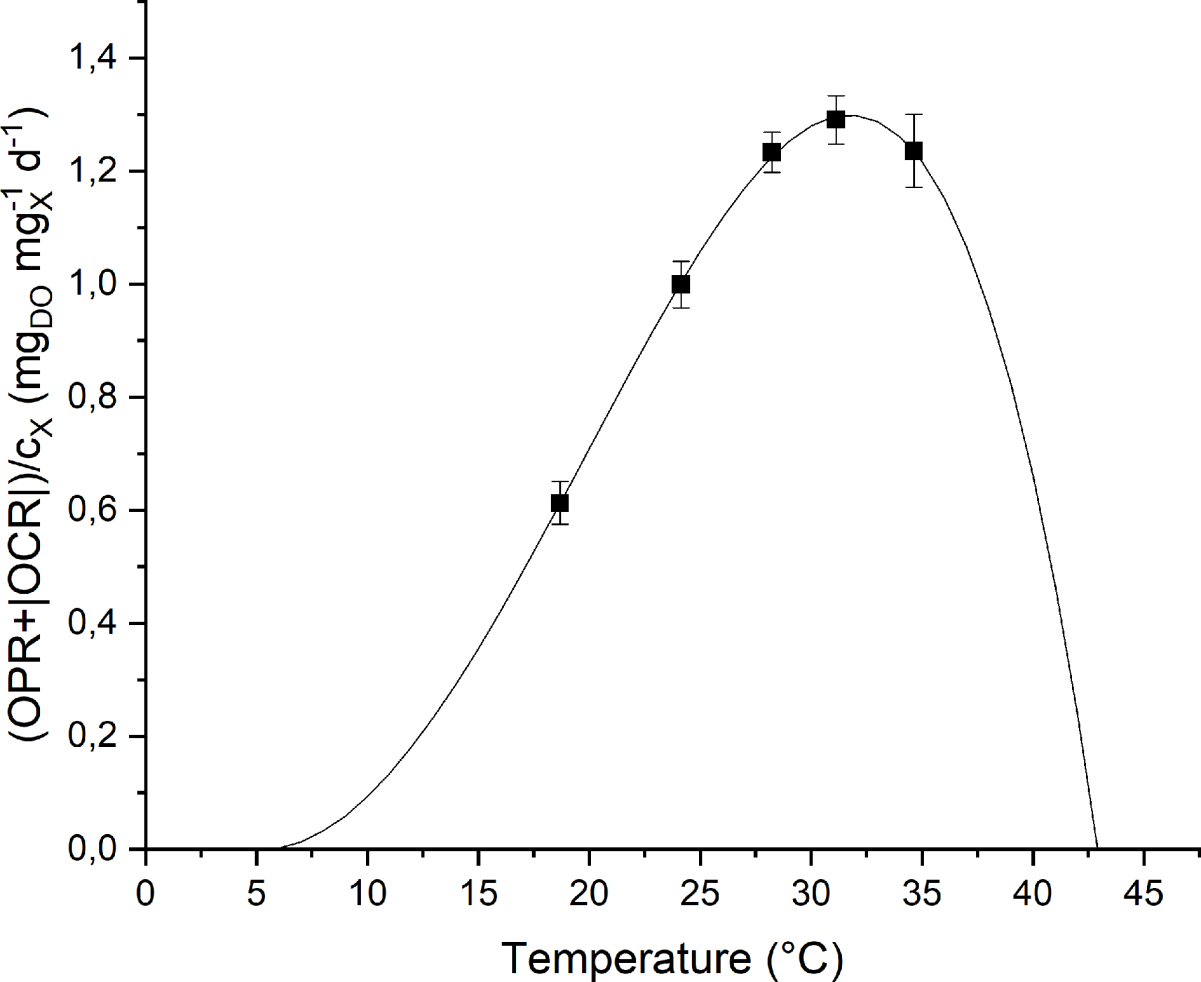
Specific oxygen production rate as a function of temperature: experimental data (dots) and fitted model (continuous line).

To study the effect of temperature on the biomass growth, all the other nutrients were supplied in excess, and the light intensity was set to its optimal value, according to the previous results. The investigated values of temperature, changed every 4 light-dark cycles, were: 18, 24, 28, 31, and 35°C. The temperature value was increased progressively by heating up the water bath. The values of *T*_*opt*_, *T*_min_ and *T*_max_ were hence fitted according to the model by Bernard and Rémond (2012) (Equation 8). Figure 6 shows the experimental data and the fitting curve.

As expected, according to Bernard and Rémond (2012), the biomass growth rate is null below *T*_min_ and above *T*_max_, and reaches its maximum value when the temperature is equal to *T*_*opt*_. In particular, for *T* > *T*_*otp*_, the growth rate rapidly decreases because of the heat stress that can affect activities of enzymes (denaturation, inactivation) or modify proteins involved in photosynthesis (Ras et al., 2013).

Moreover, it can be observed that the model (the one represented in Figure 6 by the solid line) can represent well the experimental points. The model parameters obtained from the fitting procedure together with the standard deviations are summarized in Table 3. The value found for *T*_*opt*_ is comparable to those reported in literature for microalgae: 20–30°C (Rinanti, 2016).

**Table 3:** Summary of the fitted parameters of the temperature model.

***T*_***opt***_ (^**°**^C)**	***T*_***min***_ (^**°**^C)**	**T_***max***_ (^**°**^C)**
31.74 ± 0.13	5.29 ± 1.23	42.93 ± 1.19

#### Nitrogen Effect

Since *Anabaena* PCC 7122 is a nitrogen-fixing cyanobacterium, it is interesting to study its growth in nitrogen limited conditions, in order to determine whether the concentration of atmospheric nitrogen dissolved in the liquid, as supplied in continuous cultivation by means of bubbling CO_2_-enriched air, is sufficient or limiting, and to which extent.

The values of nitrogen concentration studied, supplied as sodium nitrate and changed every 4 light-dark cycles, were: 0, 2, 6, 8, and 15 mg L^−1^. The change in nitrates concentration was obtained by progressive addition of NaNO_3_ in the sealed flask. Since *Anabaena* is not only capable of using nitrogen in nitrate form, but also atmospheric nitrogen, before each addition of NaNO_3_, the biomass sample was bubbled with argon in order to strip the dissolved atmospheric nitrogen which could alter the microorganism response to the nutrient supply.

**Figure 7:**
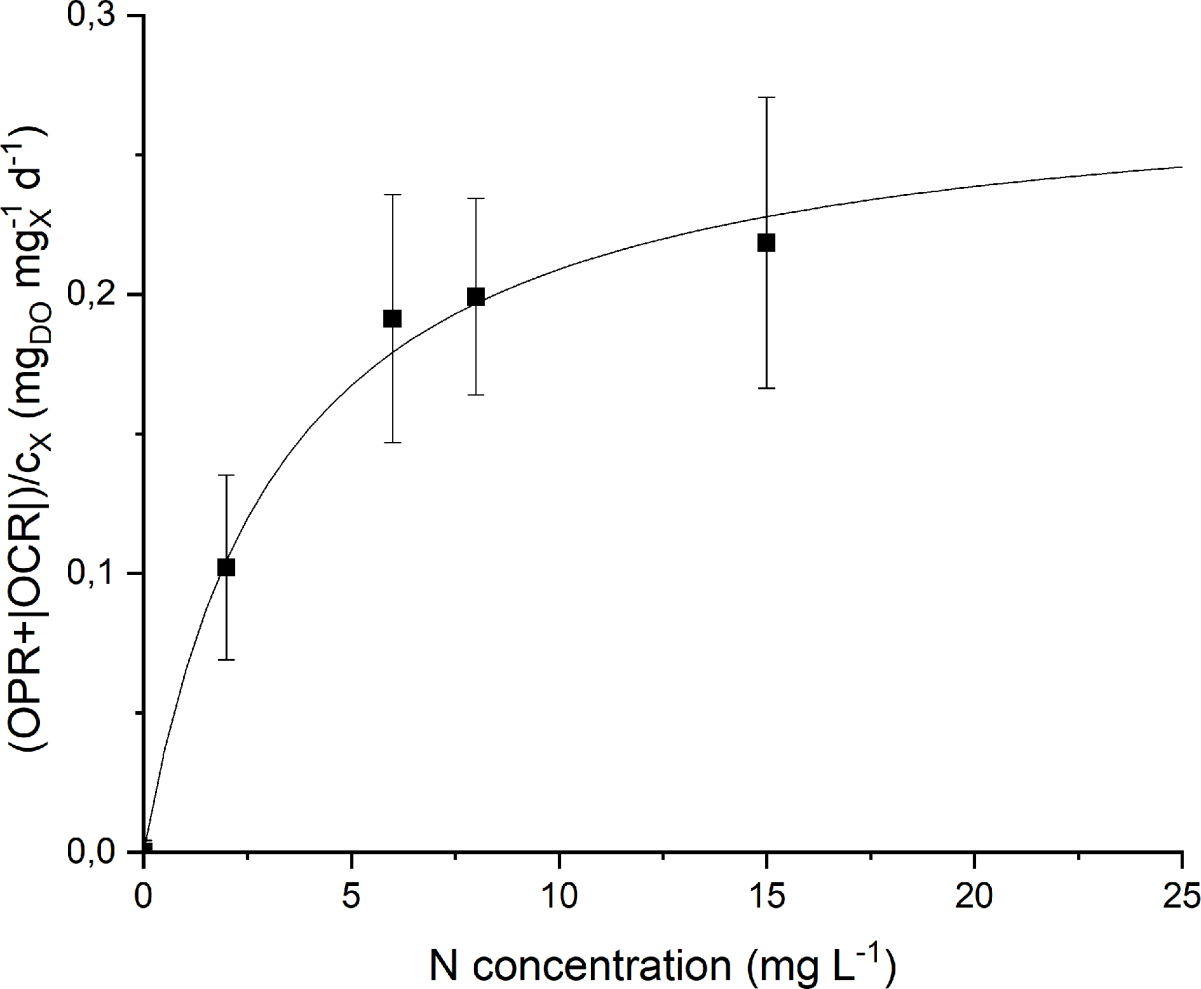
Specific oxygen production rate as a function of nitrogen concentration: experimental data (dots) and fitted Monod model (continuous line).

By analyzing the normalized OPR and OCR obtained at each nitrogen concentration (Figure S2) it could be noticed that they display a similar trend, since both of them increase (in absolute value) at increasing nitrogen concentration. Moreover, even at 0 mg L^−1^ of N and after stripping with argon, a small rate of oxygen production was detected. This can be explained considering the basal photosynthetic activity of cells: non-growing cells are responsible of a short-term oxygen production rate enhanced by the previous exposure of the preinoculum to light. This phenomenon was taken into account through a further normalization of the data, subtracting the value of $\left(\frac{OPR}{c_{x}}+\frac{\left|OCR\right|}{c_{x}}\right)$ obtained at 0 mg L^−1^ of N from all the other experimental points. Figure 7 shows the experimental results together with the fitted Monod model (Equation 9). The value of the half-saturation constant obtained from the regression resulted equal to 2.90 ± 0.67 mg_N_ L^−1^.

Note that the obtained value of *K*_*N*_ is much lower than typical values of nitrogen half-saturation constant reported in literature for green microalgae. For example, some authors measured a value of 19.4 mg L^−1^ (Rowley, 2010) and 23.4 mg L^−1^ (Ramos Tercero et al., 2014) for the green microalga *Chlorella protothecoides*. However, specific studies about cyanobacteria confirm the value obtained in our work. Cornet et al. (1992) found a half-saturation constant of 1.2 mg L^−1^ for *Spirulina platensis*, Halterman and Toetz (1984) found a value close to 0 mg L^−1^ for *Anabaena* A7214, while Hattori (1962) reported 0.98 mg L^−1^ for *Anabaena cylindrica*. This is also reasonable considering that nitrogen-fixing organisms should be adapted to the low dissolved nitrogen concentration given by the solubility of this gas in the liquid medium (~13 mg L^−1^). In this way, the supply of nitrates at higher concentrations (greater than the saturation one) does not result in improved biomass production rate, as confirmed by the results of our continuous experiments (section Continuous Cultivation).

### Maximum Specific Growth Rate **μ**_****max****_

Based on the methodology described in section 2.6, once all the kinetic parameters were determined according to the results presented, the maximum specific growth rate was calculated for each experimental condition investigated. The values obtained for each steady state of the continuous cultivation experiments is reported in Table 4. The average μ_max_ resulted equal to 8.22 ± 0.69 d^−1^.

**Table 4:** μ_max_ determination results.

**Light (μmol m^**−2**^ s^**−1**^)**	**τ (d)**	${\text{c}}_{\text{x}}^{\text{exp}}$ **(g L^**−1**^)**	**μ_max_ (d^−1^)**
190	1.32	0.47	7.49
190	1.62	0.73	8.82
190	2.5	0.93	8.51
190	2.92	0.86	7.48
190	3.68	0.91	7.22
190	4.6	1.15	8.09
650	4.1	2.41	7.95
650	2.4	2.05	8.24
650	1.9	1.90	8.50
650	1.6	1.66	8.42
650	1.0	1.40	9.66

The value obtained according to the methodology proposed here appeared slightly higher than what is usually found in the literature, where values around 2.8–3.8 d^−1^ are reported for different cyanobacteria species, such as *Synechocysits* sp. (Kim et al., 2015) and *Cyanothece* sp. (Zhang et al., 2015). On the other hand, Salleh et al. (2017) reported a value of 6 d^−1^ for *Anabaena variabilis*. It should be taken into account that this parameter represents a growth condition that can hardly occur in reality where, even if nutrients are supplied in excess, light will always play an important effect on the actual growth and respiration of the photosynthetic microorganisms. It should be noticed, in addition, that the physical meaning of such a parameter is different from that of the classical Monod function, because the dependence on light intensity is described with a Haldane-like model. Accordingly, the maximum specific growth rate for photosynthetic organisms should be described from a different perspective than for heterotrophic organisms. In fact, it represents an ideal value, useful to correctly describe the growth of the organisms from a mass balance and kinetic point of view, rather than an actual value reachable under specific growth conditions. This different approach appears reasonable when considering the complex metabolisms of photosynthetic organisms. Different metabolic pathways are in fact combined to sustain growth, as a result of complex mechanisms ranging from light capture efficiency, the ratio of the respiration rate, additional effects of light dissipation and energy losses for nitrogen fixation, that are all included in the maintenance term.

## Conclusions

In this work we propose a method to reliably determine kinetic parameters of photosynthetic microorganisms, in particular the maximum specific growth rate. Specifically, the method was applied to describe the behavior of the N-fixing cyanobacterium *Anabaena* PCC7122, whose growth rate is influenced by not only light and temperature, but may also be limited by the reduced concentration of dissolved atmospheric nitrogen. By means of well-validated respirometric tests, it was possible to measure the kinetic parameters describing the effect of light, temperature and nitrogen on the cyanobacterium growth rate. In particular, the half-saturation of N resulted lower compared to that of green microalagae, but comparable to that of other cyanobacteria. Moreover, the value of 3 mg L^−1^ found here suggests that *Anabaena* growth under N-fixing conditions is not significantly limited by atmospheric nitrogen solubility (about 13 mg L^−1^ at ambient conditions). This was also confirmed by continuous cultivation experiments, which showed only slight differences between the productivities obtained with and without additional supply of nitrates. The evaluation of the specific maintenance rate showed that this parameter is affected by the light intensity, with more energy being dissipated under high light conditions, and by the nitrogen source, with N-fixation requiring more energy for cell maintenance. Finally, the maximum specific growth rate could be determined by applying the comprehensive and fully determined kinetic model in the material balance of the continuous PBR, obtaining a constant value.

The proposed method can be easily applied to any photosynthetic microorganism as a way to quickly quantify the effect of the main process variables on the growth kinetics, and use this to perform process simulations aimed at design and control of large-scale production processes.

## Data Availability Statement

The datasets generated for this study are included in the manuscript/Supplementary Files.

## Author Contributions

EB and ES contributed the conception and design of the study. AG and LB performed the experiments. AG and EB wrote the first draft of the manuscript. EB, ES, and AG wrote the sections of the manuscript. ES and AB discussed the results. All authors contributed to manuscript revision, read, and approved the submitted version.

### Conflict of Interest

The authors declare that the research was conducted in the absence of any commercial or financial relationships that could be construed as a potential conflict of interest.

## Figures and Tables

**Table:** 

PBR = photobioreactor
*W* = width of the photobioreactor, cm
*A*_*PBR*_ = irradiated surface of the photobioreactor, cm^2^
*V*_*R*_ = volume of the photobioreactor, mL
*Q* = volumetric flow rate fed to and withdrawn from the photobioreactor, mL d^−1^
τ = hydraulic residence time, d
μ = apparent growth rate, d^−1^
μ_max_ = maximum specific growth rate, d^−1^
*OD* = optical density, abs
*c*_*x*_ = biomass concentration, g L^−1^
*P*_*x, V*_ = volumetric biomass productivity, g L^−1^ d^−1^
*P*_*x, A*_ = areal biomass productivity, g m^−2^ d^−1^
*I*_0_ = incident light intensity, μmol photons m^−2^ s^−1^
*BI* = back irradiance, μ mol photons m^−2^ s^−1^
$I_{sp}^{\exp}$ = specific light supply rate obtained from continuous cultivations, mmol photons g^−1^ d^−1^
η_*PAR*_ = photosynthetic active radiation efficiency, %
*LHV* = lower heating value of the biomass, MJ kg^−1^
*E*_*P*_ = average energy of photons, J μmol^−1^
*r*_*X*_ = biomass growth rate, mg L^−1^ d^−1^
μ_*e*_ = specific maintenance energy, d^−1^
ϕ(*T*) = limiting factor due to temperature
*T* = temperature, °C
*T*_min_ = minimum temperature (kinetic parameter), °C
*T*_max_ = maximum temperature (kinetic parameter), °C
*T*_*opt*_ = optimal temperature (kinetic parameter), °C
μ_*opt*_ = optimal growth rate at *T*_*opt*_ (kinetic parameter), d^−1^
*f*(*I*_*sp*_) = limiting factor due to specific light supply rate
*I*_*sp*_ = specific light supply rate, mmol photons g^−1^ d^−1^
*K*_*L*_ = half-saturation constant of the specific light supply rate, mmol photons g^−1^ d^−1^
*K*_*I*_ = inhibition constant of the specific light supply rate, mmol photons g^−1^ d^−1^
*f*(*N*) = limiting factor due to nitrogen
*C*_*N*_ = concentration of nitrogen, mg N L^−1^
*K*_*N*_ = nitrogen half-saturation constant, mg N L^−1^
*Y*_*O*_2_/*x*_ = yield of oxygen on biomass, mg_O2_ ${\text{mg}}_{\text{X}}^{-1}$
*k*_*L*_*a* = oxygen mass transfer coefficient, min^−1^
*OPR* = oxygen production rate, mg_O2_ L^−1^ d^−1^
*OCR* = oxygen consumption rate, mg_O2_ L^−1^ d^−1^
*C*_*O*_2__ = oxygen concentration, mg_O2_ L^−1^
$C_{O_{2}}^{*}$ = oxygen saturation concentration in the liquid, mg_O2_ L^−1^
