# Supplementary material for: Continuous Cultivation as a Method to Assess the Maximum Specific Growth Rate of Photosynthetic Organisms
Source: Front Bioeng Biotechnol. 2019 Oct 17;7:274. doi: 10.3389/fbioe.2019.00274 (PMC6811504; doi:10.3389/fbioe.2019.00274)
Supplement: Supplementary file 1 [file Table_1.DOCX]

Continuous cultivation as a method to assess the maximum specific growth rate of photosynthetic organisms

Elena Barbera^1,2^, Alessia Grandi^1^, Lisa Borella^2^, Alberto Bertucco^2^ , Eleonora Sforza^1*^

^1^Interdepartmental Centre Giorgio Levi Cases, Via Marzolo 9, 35131 Padova, Italy

^2^Department of Industrial Engineering DII, University of Padova, Via Marzolo 9, 35131 Padova, Italy

*** Correspondence:**Eleonora Sforza, PhD
[eleonora.sforza@unipd.it](mailto:eleonora.sforza@unipd.it)
Tel.: +39-0498275462; fax: +39-0498275461

Table S1: Composition of BG11 and BG11_0_ cultivation media

| **Component** | **BG11_0_** | **BG11** |
| --- | --- | --- |
|  | **(mg/L)** | **(mg/L)** |
| ***Na_2_Mg EDTA*** | 2 | 2 |
| ***FeCl_3_ ^.^ 6 H_2_O*** | 12.43 | 12.43 |
| ***Citric acid ^.^ 1 H_2_O*** | 12 | 12 |
| ***CaCl_2_ ^.^ 2 H_2_O*** | 72 | 72 |
| ***MgSO_4_ ^.^ 2 H_2_O*** | 150 | 150 |
| ***K_2_HPO_4_ ^.^ 3 H_2_O*** | 189 | 189 |
| ***Na_2_CO_3_*** | 0.04 | 0.04 |
| ***H_3_BO_3_*** | 11.44 | 11.44 |
| ***MnCl_2_ ^.^ 4 H_2_O*** | 7.24 | 7.24 |
| ***ZnSO_4_ ^.^ 7 H_2_O*** | 0.88 | 0.88 |
| ***CuSO_4_ ^.^ 5 H_2_O*** | 0.316 | 0.316 |
| ***CoCl_2_ ^.^ 6 H_2_O*** | 0.2 | 0.2 |
| ***NaMoO_4_ ^.^ 2 H_2_O*** | 1.56 | 1.56 |
| ***NaNO_3_*** | - | 3000 |
| ***NaHCO_3_*** | 2500 | 2500 |


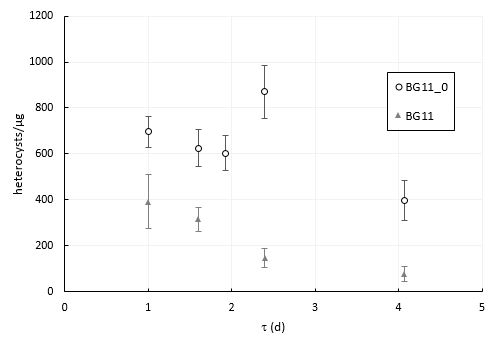


Figure S1: heterocysts concentration (n° heterocysts/μg) as a function of residence time at 650 μmol m^-2^ s^-1^ in *Anabaena* PCC7122 cultivated in BG11 (grey triangles) and BG11_0_ (open circles)


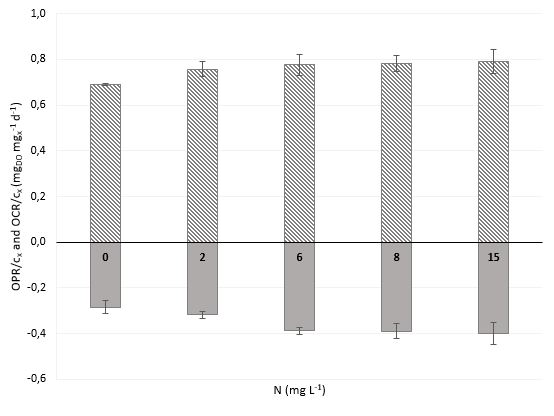


Figure S2: Specific OPR (diagonal pattern) and OCR (grey) as a function of N concentration
